# New Advances in Drug Hypersensitivity Research and Treatment

**DOI:** 10.1155/2018/9345078

**Published:** 2018-06-21

**Authors:** Yi-Giien Tsai, Wen-Hung Chung, Riichiro Abe, Wichittra Tassaneeyakul

**Affiliations:** ^1^Division of Pediatric Allergy and Immunology, Changhua Christian Hospital, Changhua City, Taiwan; ^2^School of Medicine, Kaohsiung Medical University, Kaohsiung, Taiwan; ^3^School of Medicine, Chung Shan Medical University, Taichung, Taiwan; ^4^Department of Dermatology, Drug Hypersensitivity Clinical and Research Center, Chang Gung Memorial Hospital, Taipei and Linkou, Taiwan; ^5^Chang Gung Immunology Consortium, Chang Gung Memorial Hospital, Chang Gung University, Taoyuan, Taiwan; ^6^Whole-Genome Research Core Laboratory of Human Diseases, Chang Gung Memorial Hospital, Keelung, Taiwan; ^7^College of Medicine, Chang Gung University, Taoyuan, Taiwan; ^8^Department of Dermatology, Xiamen Chang Gung Hospital, Xiamen, China; ^9^Division of Dermatology, Niigata University Graduate School of Medical and Dental Sciences, Niigata, Japan; ^10^Department of Pharmacology, Faculty of Medicine, Khon Kaen University, Khon Kaen, Thailand

Drug hypersensitivity remains an important clinical issue which is common and can be fatal with long-term complications. Severe cutaneous adverse reaction (SCAR) is T-cell-mediated delayed-type hypersensitivity, including Stevens-Johnson syndrome/toxic epidermal necrolysis (SJS/TEN), drug reactions with eosinophilia and systemic symptoms (DRESS)/drug-induced hypersensitivity syndrome (DIHS), and acute generalized exanthematous pustulosis (AGEP). These spectra of drug hypersensitivity are challenging in clinical practice and associated with the high rate of morbidity and mortality. This special issue focuses on new advances in drug hypersensitivity research and treatment. We have invited some papers to address such issues.

The first paper of this special issue provides a general overview on recent advances in the epidemiologic, genetic factors, immune mechanisms, diagnostic tools, and therapeutic approaches of drug hypersensitivity [1]. Specific immune molecules involved in SCAR, such as IL-15 in SJS/TEN or the characteristic immunohistopathological features of SJS/TEN, DRESS, and AGEP, were also reviewed in this special issue. A better illustration of the histopathological features could improve the accuracy of diagnosis and lead to give essential insight into the pathomechanism of drug hypersensitivity reactions or SCAR. This review shows an updated knowledge of drug hypersensitivity that can help clinical practice or research in this field.

To broaden our understanding of the situation of SCAR in different countries, the special issue includes epidemiologic studies of SCAR from different Asian countries, including Japan, China, and Thailand. More and more reports show anticancer drugs, especially new targeting or immune therapeutic drugs which may also cause SCAR; this special issue also includes a literature review of SJS/TEN related to anticancer drugs, including chemotherapy, targeted therapy, and immunotherapy. The rapid development of variable targeting or immunologic anticancer drugs may potentially contribute to a new threat of SCAR in the future. This article also increases clinician awareness of the differential diagnosis between immune-related hypersensitivity reactions or direct skin toxicity related to anticancer drugs that can further improve the managements of SCAR in cancer patients.

Recent advancement in pharmacogenomics reveals genetic links to SCAR. There are 3 papers in this special issue demonstrating the association between single-nucleotide polymorphisms and HLA-B alleles with adverse drug reactions, including anticonvulsant or antihyperuricemic agent-induced hypersensitivity reactions or drug-induced liver injury. Furthermore, different techniques used to screen HLA alleles or predict drug hypersensitivity reactions in new drug users were also reviewed in one paper.

There is still no consensus-specific treatment for SCAR. Due to the rarity of SCAR, there were only few well-designed and implemented large-scale randomized control trials of treatment for patients with SCAR. Systemic corticosteroid is still controversial for the management of SJS/TEN. There are more evidences showing beneficial therapeutic effects of cyclosporine and biologic anti-TNF alpha blockade on patients with SJS/TEN. In this special issue, one paper gives a concise review on the management of each SCAR based on current clinical evidences.

## References

[1] Chen C. B. , Abe R., Pan R. Y. (2018). An updated review of the molecular mechanisms in drug hypersensitivity. *Journal of Immunology Research*.

